# Diagnostic value of serum levels of malondialdehyde (MDA), superoxide dismutase (SOD), glutathione peroxidase (GSH-PX), IL-6, TNF-a, and IL-1b in preserving kidney function in diabetic renal cell carcinoma patients undergoing partial resection: A focus on the TGF-b1/Smad pathway

**DOI:** 10.5937/jomb0-55290

**Published:** 2025-09-05

**Authors:** Pengcheng Ye, Jia Fu

**Affiliations:** 1 Peking University First Hospital, Department of Anesthesiology, Beijing, 100034, China; 2 Beijing Fengtai Hospital, Department of Anesthesiology, Beijing, 100071, China

**Keywords:** dexmedetomidine, renal damage, diabetic nephropathy, renal cell carcinoma, partial nephrectomy, oxidative stress, MDA, SOD, GSH-Px, IL-6, TNF-a, TGF-b1/Smad pathway, renal function, apoptosis, deksmedetomidin, oštećenje bubrega, dijabetička nefropatija, bubrežni karcinom, parcijalna nefrektomija, oksidativni stres, MDA, SOD, GSH-Px, IL-6, TNF-a, TGF-b1/Smad putanja, funkcija bubrega, apoptoza

## Abstract

**Background:**

This study aimed to evaluate the protective effects of dexmedetomidine (DEX) on renal damage in diabetic patients with renal cell carcinoma undergoing partial nephrectomy. Specifically, it focused on oxidative stress markers, inflammatory cytokines, and the TGF-b1/Smad signalling pathway.

**Methods:**

Between August 2022 and July 2024, 100 diabetic patients with renal cell carcinoma undergoing partial nephrectomy were randomly assigned to receive either 1.0 mg/kg of DEX or normal saline (control) during surgery. Blood samples were taken preoperatively, intraoperatively, and postoperatively to measure oxidative stress markers (malondialdehyde [MDA], superoxide dismutase [SOD], glutathione peroxidase [GSH-Px]), inflammatory cytokines (IL-6, TNF-a, IL-1b), and renal function indicators (BUN, Scr, CysC). The TGF-b1/Smad pathway and renal cell apoptosis were also assessed.

**Results:**

No significant differences in baseline markers were observed between the groups. However, during and after surgery, the DEX group exhibited significantly lower MDA levels and higher SOD and GSH-Px levels (P<0.05), indicating reduced oxidative stress. Inflammatory markers (IL-6, TNF-a, IL-1b) were also lower in the DEX group.

**Conclusions:**

DEX significantly mitigates renal damage in diabetic patients undergoing partial nephrectomy by reducing oxidative stress, inflammation, and apoptosis and by inhibiting the TGF-b1/Smad pathway. These findings suggest its potential as a protective agent for high-risk surgical patients.

## Abstract (sr)

**Uvod:**

Cilj ove studije bio je da se procene zaštitni efekti deksmedetomidina (DEX) na oštećenje bubrega kod pacijenata dijabetičara sa bubrežnim karcinomom koji se podvrgavaju parcijalnoj nefrektomiji. Konkretno, fokusirala se na markere oksidativnog stresa, inflamatorne citokine i TGF-b1/Smad signalnu putanju.

**Metode:**

Između avgusta 2022. i jula 2024. godine, 100 pacijenata dijabetičara sa bubrežnim karcinomom koji su se podvrgavali parcijalnoj nefrektomiji nasumično je raspoređeno da prime ili 1,0 mg/kg DEX ili fiziološki rastvor (kontrolna grupa) tokom operacije. Uzeti su uzorci krvi preoperativno, intraoperativno i postoperativno kako bi se merili markeri oksidativnog stresa (malondialdehid [MDA], superoksid dismutaza [SOD], glutation peroksidaza [GSH-Px]), inflamatorni citokini (IL-6, TNF-a, IL-1b) i pokazatelji funkcije bubrega (BUN, Scr, CysC). Takođe su procenjeni TGF-b1/Smad putanja i apoptoza bubrežnih ćelija.

**Rezultati:**

Nisu primećene značajne razlike u osnovnim markerima između grupa. Međutim, tokom i nakon operacije, DEX grupa je imala značajno niže nivoe MDA i više nivoe SOD i GSH-Px (P<0,05), što ukazuje na smanjenje oksidativnog stresa. Inflamatorni markeri (IL-6, TNF-a, IL-1b) takođe su bili niži u DEX grupi (P<0,05).

**Zaključak:**

DEX značajno ublažava oštećenje bubrega kod pacijenata dijabetičara koji se podvrgavaju parcijalnoj nefrektomiji smanjenjem oksidativnog stresa, inflamacije i apoptoze, kao i inhibicijom TGF-b1/Smad putanje. Ovi rezultati sugerišu njegov potencijal kao zaštitnog agensa za pacijente sa visokim rizikom tokom hirurških intervencija.

## Introduction

The incidence of diabetes steadily rises with advancing age and an increasingly obese population [1]. This disease primarily results from elevated blood glucose levels due to insufficient insulin secretion. As a result, diabetes has become recognised as a global epidemic, rapidly escalating and threatening the health and quality of life of those affected [2]. Both diabetes and cancer are chronic inflammatory conditions. Despite effective blood glucose management, diabetes continues to drive chronic inflammation, which significantly heightens the risk of developing renal cell carcinoma (RCC) [3]. Notably, individuals aged 50 to 70 show the highest incidence and least favourable outcomes regarding RCC [4].

At present, clinical practice lacks sufficient medications for effectively treating RCC. Surgical resection remains a crucial therapeutic option, with radical surgery offering potential cures for patients diagnosed with early-stage RCC [5]. However, surgery, while beneficial, can also trigger inflammatory responses, increasing levels of IL-6, TNF-α, and IL-1β and altering the balance of antioxidants such as MDA, SOD, and GSH-Px due to anaesthesia-induced stress reactions. These changes can impair kidney function, leading to renal damage. Furthermore, diabetes itself, through sustained high glucose levels, generates free radicals, which reduce antioxidant capacity, exacerbate renal injury, and increase the risk of renal failure, thereby raising patient mortality [6]. The incidence of diabetes in RCC patients undergoing surgery is over 20% [7]. Long-term poor blood glucose control during surgery compromises immune function, impairs wound healing, causes endothelial cell dysfunction, intensifies the stress response, and amplifies damage to renal tissue [8]. As a result, patients with diabetic RCC undergoing surgery are at a heightened risk for renal injury. Therefore, it is crucial to identify optimal anaesthetic agents that can reduce metabolism, mitigate the stress response, and protect renal function during surgical treatment.

The TGF-β1/Smad signalling pathway is central to developing diabetic nephropathy (DN). TGF-β1 is a key factor in the progression of DN and serves as both a critical therapeutic target and an important biomarker for chronic renal diseases in clinical settings [9]. Through its interaction with the Smads2 and Smads3 receptors, TGF-β1 promotes renal fibrosis, podocyte injury, and the accumulation of extracellular matrix [10]. Elevated expression of Smads2 and Smads3 accelerates renal fibrosis [11]. Research indicates that activating the TGF-β1/Smad pathway produces advanced glycation end products, contributing to renal damage. In contrast, inhibiting this pathway is essential for reducing renal injury in diabetic RCC patients after surgery [12].

Dexmedetomidine (DEX), a selective α2-adrenergic receptor agonist, is commonly used as an adjunct in clinical anaesthesia. Beyond its role in maintaining anaesthesia, DEX offers protective effects through its antioxidant, anti-inflammatory, and anti-apoptotic properties, especially in preventing organ ischemia-reperfusion injury [13]. DEX has been shown to safeguard renal function, reduce stress responses, inhibit inflammation, and improve outcomes in organ and tissue ischemia-reperfusion injuries [14].

In the context of diabetes, a study was conducted to assess the impact of DEX on the TGF-β1/Smad signalling pathway and its protective role on renal function in patients with diabetic RCC undergoing partial nephrectomy. The findings from this investigation are discussed below.

## Materials and methods

### Data

Between August 2022 and July 2024, our institution enrolled 100 patients suffering from renal injury secondary to diabetic nephropathy for partial nephrectomy. Participants were divided into a control group and a research group of fifty according to the type of anaesthesia used. The control group consisted of 31 males and 19 females, aged between 45 and 64 years, with a mean age of 57.31±6.29 years. The length of illness ranged from 2 to 10 years, averaging 6.37±2.59 years. The BMI of patients was between 20 and 28 kg/m^2^, averaging 24.38±3.47 kg/m^2^. Fasting plasma glucose levels ranged from 6 to 10 mmol/L, averaging 8.99±1.16 mmol/L; 2-hour postprandial glucose levels were between 8 and 14 mmol/L. The average concentration was (11.35±2.67) mmol/L. Systolic blood pressure (SBP): 130–172 mmHg, mean SBP: (159.85±10.75) mmHg; diastolic blood pressure (DBP): 68–97 mmHg, mean DBP: (88.17±6.29) mmHg; heart rate (HR): 65–86 beats/min, mean HR: (73.68±4.09) beats/min. The research group comprised 33 males and 17 females, aged between 47 and 63 years, with a mean age of 57.50±6.19 years. Their illness duration extended from 3 to 9 years, with an average duration of 6.01±2.07 years. Body Mass Index (BMI) values varied from 21 to 27 kg/m^2^, averaging 24.04±3.19 kg/m^2^. Fasting plasma glucose (FPG) levels were between 7 and 11 mmol/L, averaging 8.34±1.07 mmol/L; two-hour postprandial glucose levels ranged from 9 to 15 mmol/L, with an average of 11.42±2.87 mmol/L. Systolic blood pressure (SBP) varied from 132 to 170 mmHg, with an average of 159.32±10.38 mmHg; diastolic blood pressure (DBP) was between 69 and 95 mmHg, averaging 88.00±6.02 mmHg; heart rate (HR) spanned from 66 to 85 beats per minute, with an average of 73.17±4.22 beats per minute. No significant differences were observed in the baseline data of the two groups (P>0.05).

### Inclusion and exclusion criteria

Inclusion criteria include: (A) Confirmation of diabetic nephropathy and renal injury via lab testing; (B) UAER over 30 mg/24 hours or a UACR exceeding 30 mg/g; a GFR below 60 mL/min/1.73m^2^; (C) Under going initial surgical intervention at our facility; (D) Normal cognitive abilities, mental health, and communicative competence in patients.

Exclusion criteria include: (A) Previous history of severe allergy to drugs; (B) Long-term application of analgesics or hypnotics; (C) Severe hepatic and renal failure; (D) Combined with chronic and acute infections; (E) Combined with fat metabolising diseases. Patients receive information about the study and provide their consent by signing an informed consent document.

### Methods

Research group: DEX was used to maintain anaesthesia. Before anaesthesia induction, DEX (GYZZ: Manufacturer: Specification:) 1.0 μg/kg was intravenously infused, then DEX μg/(kg·h) was selectedfor maintenance intravenous infusion until 30 min before the end of surgery.

Control group: Normal saline was used to maintain anaesthesia. 0.9% Sodium Chloride Injection was infused in equal volumes in the same way.

### Observation indicators

Obtaining test samples: Take venous blood from the patient’s elbow and allow it to stand at room temperature for 2 hours. Centrifuge the sample at 4°C, using a 10 cm centrifugation radius and a speed of 3000 revolutions per minute for two cycles, each lasting 15 minutes. After obtaining supernatant, divide the samples into 3 portions and store them at -20°C. During the surgery, normal renal tissue (5 cm away from the lesion) was removed to take 3×3×3 cm test samples and stored in 4% formaldehyde solution. On Day 2, the tissue samples were dehydrated using concentration gradient sucrose and then paraffin-embedded to form 5 mm paraffin sections. Measurements of oxidative stress, kidney function, and the TGFb1/ Smad pathway were taken before anaesthesia (T_0_), throughout the surgery (T_1_), and at surgery’s conclusion (T_1_). After tissue samples were obtained after surgery, apoptosis and apoptotic protein levels in renal tissue were assessed.

2 mL of serum was extracted to measure MDA, SOD, and GSH-Px levels using ELISA kits: MDA with the *MDA Assay Kit* (Cayman Chemical, Catalog No. 10009055), SOD with the *Superoxide Dismutase (SOD)* *Activity Assay Kit* (Abcam, Catalog No. ab65354), and GSH-Px with the *Glutathione Peroxidase Assay Kit* (Cayman Chemical, Catalog No. 703102). For inflammatory markers, IL-6, IL-1β, and TNF-α were measured with the *Human IL-6 ELISA Kit *(R&D Systems, Catalog No. DY206), *Human IL-1β ELISA Kit* (BioLegend, Catalog No. 431304), and *Human TNF-alpha ELISA Kit* (ThermoFisher, Catalog No. BMS223). For renal function, serum samples were stored at 4°C and equilibrated at room temperature for 30 minutes before measuring CysC with the *Cystatin C ELISA Kit* (BioVision, Catalog No. K1292-100), BUN with the *Blood Urea Nitrogen Assay Ki*t (Abcam, Catalog No. ab83362), and sCr with the *Serum Creatinine Assay Kit* (Sigma-Aldrich, Catalog No. MAK080).

TGF-β1/Smad pathway: Samples of tissue were obtained to analyse TGF-β1, Smad2 mRNA levels, and Smad3 mRNA in renal tissue via Western blot methods.

Assessment of apoptosis and apoptotic indicators: Tissue samples were first dewaxed and rehydrated using xylene and alcohol, then treated with a 3% hydrogen peroxide-methanol solution and TdT enzyme reaction mixture. These were incubated at 37°C for one hour within a humidified chamber. After the reaction, the samples underwent four washes with phosphate-buffered saline and were stained with diaminobenzidine for five minutes. After staining, the samples were dehydrated thrice with xylene, air-dried, and sealed for microscopic examination to assess apoptosis rates. RIPA lysate was used to extract protein from renal tissue, and a BCA assay determined the protein concentration. Subsequently, the proteins were separated by 10% SDS-PAGE and transferred to a polyvinylidene fluoride (PVDF) membrane using a transfer apparatus. The membrane was then blocked with 5% skim milk for two hours at ambient temperature before overnight incubation with primary antibodies (Cleaved Caspase-3 at 1:900; TGF-β at 1:1100; phosphorylated Smad3 at 1:900; Ki-67 at 1:1000; phosphorylated Smad2 at 1:1000) at 4°C. Secondary antibodies were applied the next day to attach to any remaining primary antibodies, with incubation continuing for one hour at room temperature. Enhanced chemiluminescence (ECL) was used for colour development, followed by exposure in a dark room for image capture.

### Statistical analysis

Two individuals conducted data entry, and the data were analysed using SPSS software version 25.0. Continuous data that followed a normal distribution were expressed as the mean ± standard deviation (x̄±s). Categorical data were presented as case numbers (n) and percentages (%). To analyse the continuous data, the t-test was used to compare the means between two groups, while the χ^2^ test (Chi-square test) was applied to analyse categorical data and assess associations between variables. A p-value of less than 0.05 was considered statistically significant, indicating that any observed differences were unlikely to be due to random chance.

## Results

### Comparative analysis of oxidative stress markers among the groups

Before administering anaesthesia, oxidative stress markers were assessed across the groups, with no significant variations observed (P>0.05). During and after the operation, MDA levels in the research group were lower, while SOD and GSH-Px levels were elevated when compared with the control group (P<0.05). For more detailed results, see Table 1.

**Table 1:** Comparative analysis of oxidative stress markers among the groups (x̄±s).

Group	n	MDA (mmol/L)			SOD (U/mL)			GSH-Px (U/mL)		
		T_0_	T_1_	T_2_	T_0_	T_1_	T_2_	T_0_	T_1_	T_2_
Control group	50	6.29±<br>0.10	8.35±<br>1.54	9.46±<br>1.09	79.36±<br>9.52	84.89±<br>10.33	88.15±<br>9.36	205.37±<br>12.09	215.76±<br>13.90	219.46±<br>14.29
Research group	50	6.30±<br>0.08	7.98±<br>1.23	8.54±<br>0.92	79.29±<br>9.14	80.48±<br>10.06	83.19±<br>8.42	205.46±<br>12.34	208.69±<br>13.51	213.29±<br>13.60
*t*		0.552	9.684	4.520	0.038	2.163	2.786	0.037	2.579	2.212
*P*		0.582	<0.05	<0.05	0.970	0.033	0.006	0.971	0.011	0.029

### Evaluation of inflammatory indices across the groups

Before anaesthesia was administered, an evaluation of inflammatory markers showed no notable differences among the groups (P>0.05). Yet, throughout and following the surgery, IL-6, TNF-α, and IL-1β levels were observed to be reduced in the research group relative to the control group (P<0.05). More comprehensive data can be found in Table 2.

**Table 2:** Evaluation of inflammatory indices across the groups (x̄±s).

Group	n	IL-6 (pg/mL)			TNF-α (ng/mL)			IL-1β (ng/mL)		
		T_0_	T_1_	T_2_	T_0_	T_1_	T_2_	T_0_	T_1_	T_2_
Control group	50	15.06±<br>1.68	19.68±<br>2.05	24.91±<br>2.48	8.26±<br>1.17	10.37±1.	15.86±<br>2.49	28.49±<br>3.15	43.29±<br>4.19	51.96±<br>5.72
Research group	50	15.19±<br>1.47	17.29±<br>1.86	22.09±<br>2.49	8.31±<br>1.19	9.58±<br>1.23	13.48±<br>2.37	28.51±<br>3.60	38.45±<br>4.06	45.29±<br>4.82
*t*		0.412	6.105	5.674	0.212	3.134	4.896	0.030	5.866	6.305
*P*		0.681	<0.05	<0.05	0.833	0.002	<0.05	0.977	<0.05	<0.05

### Assessment of TGF-β1/Smad pathway markers between groups

**Figure 1:**
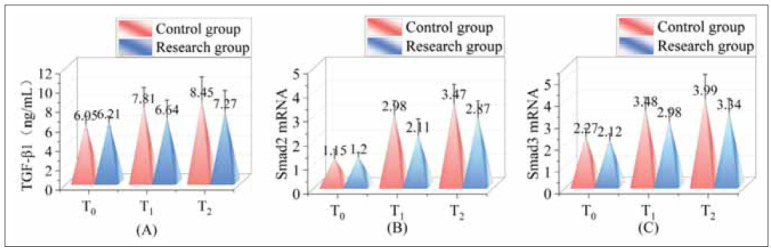
Assessment of TGF-β1/Smad pathway markers between groups.<br>Note: (A) is TGF-β1, (B) is Smad2 mRNA and (C) is Smad3 mRNA; T_0_ indicates before anaesthesia, T_1_ indicates during surgery, and T_2_ indicates the end of surgery.

Before anaesthesia, the TGF-β1/Smad pathway markers were analysed between the two research groups, with no significant variance found (P>0.05). After anaesthesia, the concentrations of TGF-β1, Smad2 mRNA, and Smad3 mRNA were reduced in the research group compared to the control group (P<0.05). Comprehensive results are depicted in Figure 1.

### Analysis of renal function indices across both group

**Figure 2:**
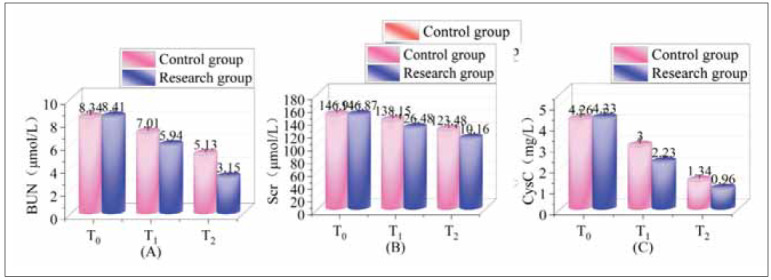
Analysis of renal function indices across both groups.<br>Note: (A) is BUN, (B) is Scr, and (C) is CysC; T_0_ indicates before anaesthesia, T_1_ indicates during surgery, and T_2_ indicates the end of surgery.

Before administering anaesthesia, renal function parameters were assessed across both groups, showing no notable disparities (P>0.05). Throughout the surgery and in the postoperative period, BUN, Scr, and CysC measurements were lower in the research group than in the control group (P<0.05). Further details are available in Figure 2.

### Comparison of apoptosis between two groups before anaesthesia

Within the research group, both the apoptosis rate and the concentrations of Cleaved Caspase-3 in renal tissues were observed to be reduced relative to the control group, while Ki-67 levels were elevated. For detailed comparisons, see Table 3.

**Table 3:** Comparison of renal cell apoptosis between the two groups after anaesthesia (n, %).

Group	n	Apoptosis rate of renal tissue (%)	Cleaved Caspase-3 (μmol/L)	Ki-67 (%)
Control group	50	13.49±1.25	33.58±1.87	10.67±2.07
Research group	50	8.28±0.36	21.39±1.34	12.68±3.49
*t*		28.321	37.468	3.503
*P*		<0.05	<0.05	0.001

## Discussion

Clinical pharmacology studies have demonstrated that when used as an adjuvant to anaesthesia, DEX serves its anaesthetic purpose and exerts a range of beneficial effects, including anti-inflammatory, antioxidant, anticancer, and blood flow regulation properties [15]. DEX can modulate the p38MAPK/TXNIP signalling pathway, suppress the release of inflammatory cytokines, reduce oxidative damage, minimise apoptosis in nerve cells, and alleviate ischemia-reperfusion injury in organs and tissues [16]. Additionally, DEX’s antioxidative properties help relieve ischemia-reperfusion damage, with its preoperative administration playing a crucial role in reducing inflammatory responses and improving recovery from renal ischemia [17]. Despite these known benefits, there is limited research on the application of DEX in surgical patients with diabetic renal cell carcinoma. Thus, further exploration into the mechanisms by which DEX improves renal damage in patients undergoing surgery is necessary.

Chronic hyperglycemia in diabetes leads to an increase in reactive oxygen species (ROS), which play a pivotal role in the complications associated with diabetes [2]. These ROS can oxidise cellular proteins, DNA, lipids, and carbohydrates, exacerbating diabetic complications [18]. In diabetic patients, renal cell carcinoma (RCC) emerges as a serious complication, with oxidative stress in a high-glucose environment contributing to cancer cell proliferation [19]. The imbalance between antioxidant enzymes and oxidative molecules is primarily responsible for oxidative damage. Excessive production of reactive oxides diminishes the protective capacity of antioxidants [20]. Studies have shown that as diabetic renal cell carcinoma progresses, there is an increase in malondialdehyde (MDA), a byproduct of lipid peroxidation, while antioxidants such as superoxide dismutase (SOD) and glutathione peroxidase (GSH-Px) decrease significantly [21]. Notably, research indicates that DEX can improve the levels of these antioxidants (SOD, GSH-Px) and reduce MDA levels in patients undergoing surgery for diabetic renal cell carcinoma.

DEX, a highly selective α2-adrenergic receptor agonist, can modulate the concentration of cyclic adenosine monophosphate (cAMP) in cells, decrease norepinephrine release, inhibit sympathetic nervous activity, and exert a sedative effect. This action helps maintain hemodynamic stability, reduces sympathetic nervous system excitability, and mitigates the traumainduced stress response during surgery for diabetic renal cell carcinoma. Furthermore, DEX’s prolonged half-life and high protein-binding affinity enhance its pharmacologic effects, which include suppressing pain signal transmission, regulating catecholamines, and stimulating vagus nerve activity, thereby providing significant analgesia. Studies by Zhang et al. [16] have shown that DEX can alleviate apoptosis in liver ischemia-reperfusion injury by reducing oxidative stress. However, sustained high glucose levels in diabetes contribute to systemic oxidative stress, and maintaining stable blood glucose during surgery is critical to minimising oxidative damage [22].

Surgical trauma, including tracheal intubation, manipulation, and skin incision, can provoke an inflammatory response in patients with diabetic renal cell carcinoma. Inflammatory factors can compromise the renal capillary barrier, leading to renal dysfunction and tissue damage [23]. Tumour necrosis factor-alpha (TNF-α), a proinflammatory cytokine, is synthesised in response to physical trauma or infection and activates the release of other cytokines like IL-6 and IL-1β, thereby promoting inflammation [24]. Studies have demonstrated that DEX significantly reduces TNF-α, IL-6, and IL-1β levels in patients undergoing surgery for diabetic renal cell carcinoma (P<0.05). This indicates that DEX effectively mitigates the inflammatory response, thus preventing inflammation-induced renal damage. The mechanism behind this anti-inflammatory effect likely involves the strong stimulation of α2 receptors, which suppress norepinephrine release and inhibit pain signal transmission. Further studies have also supported the notion that DEX alleviates renal damage in various contexts, including sepsis [25], endotoxic shock [26], and neuroinflammation [27], though the exact mechanisms underlying DEX’s anti-inflammatory actions remain unclear and warrant further investigation.

The potential role of inflammatory markers in diabetic kidney disease (DKD) has been emphasised by Khanijou et al. (28), who reviewed biomarkers like TNFR-1 and kidney injury molecule-1 (KIM-1) for their potential in early detection and monitoring of DKD. This aligns with findings from our study, which shows that DEX reduces oxidative stress, inflammation, and apoptosis in diabetic patients undergoing partial nephrectomy, suggesting that DEX has a protective role in kidney injury. Both studies highlight the importance of inflammation and oxidative stress in DKD, with Khanijou et al. [28] focusing on biomarkers for diagnosis, while our research illustrates how therapeutic interventions like DEX can mitigate kidney damage and improve patient outcomes.

Our research also explored the effect of DEX on the TGF-β1/Smad signalling pathway, finding that markers associated with this pathway were significantly reduced in the DEX-treated group compared to the control group (P<0.05). The TGF-β1/Smad pathway regulates inflammation and oxidative stress, playing a key role in developing renal injury after surgery in diabetic renal cell carcinoma patients [28]. Previous studies, have shown that reducing TGF-β1 expressioncan slow the progression of renal fibrosis [29]. DEX has effectively reduced the activation of the TGF-β1/Smad pathway induced by surgery and anaesthesia, lowering TGF-β1, Smad2, and Smad3 levels and offering potential therapeutic benefits in preventing renal fibrosis.

Diabetes exacerbates renal dysfunction by disrupting glucose metabolism and affecting renal blood flow, leading to the accumulation of reactive oxides and fibrosis in the glomerulus and tubulointerstitium [30]. Surgical trauma, anaesthesia, and inflammatory responses during surgery further aggravate renal tissue damage, contributing to a decline in renal function [31]. Our study found that DEX treatment significantly reduced apoptosis rates and Cleaved Caspase-3 levels in renal tissues compared to the control group, while Ki-67 levels, a cell proliferation marker, were increased (P<0.05). These findings suggest that DEX helps minimise oxidative damage and tissue injury by scavenging free radicals and superoxides generated in renal cells during surgery. Moreover, DEX reduces the release of inflammatory cytokines and alleviates oxidative stress, regulating the TGF-β1/Smad pathway and improving renal tissue function. These results are consistent with findings by Lankadeva et al., who demonstrated that DEX reduces norepinephrine requirements, preserves renal medullary function, and mitigates renal damage [32].

A key limitation of this study was its small sample size, which was drawn from a single centre, potentially limiting the generalizability of the findings. This restriction may have introduced variability and bias, making applying the results to a broader population difficult. Additionally, the study lacked long-term follow-up to monitor patients’ recovery, post-surgery complications, and any potential adverse effects of dexmedetomidine (DEX) over time. Without extended monitoring, the long-term benefits and safety of DEX in diabetic renal cell carcinoma patients remain uncertain. Future studies with larger multicenter samples and longer follow-up periods would provide more reliable and comprehensive data on DEX’s effects.

In conclusion, DEX significantly enhances renal function in patients undergoing surgery for diabeticrenal cell carcinoma by delaying apoptosis, reducing oxidative stress, and modulating the TGF-β1/Smad signalling pathway. DEX also offers protection against renal tissue damage caused by disease, surgery, and anaesthesia. However, several limitations exist, including the small sample size and the single-centre nature of the study, which may lead to variability in outcomes. The study did not include long-term monitoring to track recovery and potential adverse events post-surgery. Future research should aim to expand the sample size, conduct multicenter studies, and extend follow-up periods to provide a more comprehensive understanding of DEX’s efficacy and its potential to improve patient outcomes in diabetic renal cell carcinoma surgery [33].

## Dodatak

### Acknowledgements

We thank the patients who participated in this study and the medical staff at Peking University First Hospital and Beijing Fengtai Hospital for their support in patient recruitment and care. Special thanks to the technical teams at both institutions for their assistance in data collection and analysis.

### Funding

This study received no funding.

### Ethical approval

The study was approved by the Institutional Review Boards of Peking University First Hospital and Beijing Fengtai Hospital, and informed consent was obtained from all participants.

### Data availability

Data are available from the corresponding author upon reasonable request.

### Conflict of interest statement

All the authors declare that they have no conflict of interest in this work.
